# Deforestation effects on *Attalea* palms and their resident *Rhodnius*, vectors of Chagas disease, in eastern Amazonia

**DOI:** 10.1371/journal.pone.0252071

**Published:** 2021-05-20

**Authors:** Walter Souza Santos, Rodrigo Gurgel-Gonçalves, Lourdes Maria Garcez, Fernando Abad-Franch

**Affiliations:** 1 Laboratório de Epidemiologia das Leishmanioses, Instituto Evandro Chagas, Secretaria de Vigilância em Saúde, Ministério da Saúde, Ananindeua, Pará, Brazil; 2 Programa de Pós-graduação em Biologia Parasitária na Amazônia, Universidade do Estado do Pará, Belém, Pará, Brazil; 3 Núcleo de Medicina Tropical, Faculdade de Medicina, Universidade de Brasília, Brasília, Distrito Federal, Brazil; 4 Laboratório de Parasitologia Médica e Biologia de Vetores, Faculdade de Medicina, Universidade de Brasília, Brasília, Distrito Federal, Brazil; 5 Departamento de Patologia, Universidade do Estado do Pará, Belém, Pará, Brazil; 6 Grupo Triatomíneos, Instituto René Rachou–Fiocruz, Belo Horizonte, Minas Gerais, Brazil; Fundacao Oswaldo Cruz, BRAZIL

## Abstract

*Attalea* palms provide primary habitat to *Rhodnius* spp., vectors of *Trypanosoma cruzi*. Flying from palms, these blood-sucking bugs often invade houses and can infect people directly or via food contamination. Chagas disease (CD) risk may therefore increase when *Attalea* palms thrive near houses. For example, *Attalea* dominate many deforested landscapes of eastern Amazonia, where acute-CD outbreaks are disturbingly frequent. Despite this possible link between deforestation and CD risk, the population-level responses of Amazonian *Attalea* and their resident *Rhodnius* to anthropogenic landscape disturbance remain largely uncharted. We studied adult *Attalea* palms in old-growth forest (OGF), young secondary forest (YSF), and cattle pasture (CP) in two localities of eastern Amazonia. We recorded 1856 *Attalea* along 10 transects (153.6 ha), and detected infestation by *Rhodnius* spp. in 18 of 280 systematically-sampled palms (33 bugs caught). Distance-sampling models suggest that, relative to OGF, adult *Attalea* density declined by 70–80% in CP and then recovered in YSF. Site-occupancy models estimate a strong positive effect of deforestation on palm-infestation odds (β_CP-infestation_ = 4.82±1.14 SE), with a moderate decline in recovering YSF (β_YSF-infestation_ = 2.66±1.10 SE). Similarly, *N*-mixture models suggest that, relative to OGF, mean vector density sharply increased in CP palms (β_CP-density_ = 3.20±0.62 SE) and then tapered in YSF (β_YSF-density_ = 1.61±0.76 SE). Together, these results indicate that disturbed landscapes may support between ~2.5 (YSF) and ~5.1 (CP) times more *Attalea*-dwelling *Rhodnius* spp. per unit area than OGF. We provide evidence that deforestation may favor palm-dwelling CD vectors in eastern Amazonia. Importantly, our landscape-disturbance effect estimates explicitly take account of (i) imperfect palm and bug detection and (ii) the uncertainties about infestation and vector density arising from sparse bug data. These results suggest that incorporating landscape-disturbance metrics into the spatial stratification of transmission risk could help enhance CD surveillance and prevention in Amazonia.

## Introduction

Chagas disease remains a major public-health concern across Latin America and, to a lesser extent, the southern United States [1, 2]. Tens of thousands of people still get infected with *Trypanosoma cruzi* each year, mainly through direct or indirect contact with parasite-carrying vectors–a group of 150+ species of blood-sucking bugs known as triatomines [1, 3, 4]. Wild populations of locally native triatomine-bug species are involved in most such transmission events [5, 6]. For example, palm-dwelling bugs of the genus *Rhodnius* often invade, and sometimes breed in, human dwellings across southern Mesoamerica [7], northern South America including Colombia and Ecuador [8, 9], the Orinoco basin [10, 11], the Cerrado and Caatinga of central-northeastern Brazil [12–14], and Amazonia [14, 15].

Large-crowned palms of the genus *Attalea* provide primary habitat to wild *Rhodnius* populations in many forested, rural, and suburban landscapes across the Amazon basin [16–19]. Flying from the palm-crowns they breed in, adult (i.e., winged) bugs enter houses and other premises, perhaps attracted to artificial light [20], and can transmit *T*. *cruzi* to people either directly or via food contamination [21, 22]. Chagas disease transmission risk is therefore thought to be higher in disturbed landscapes where *Attalea* palms are abundant near houses [17, 18, 21, 22]. For example, *Attalea* palms dominate many rural landscapes of eastern Amazonia–the region from which the vast majority of acute Chagas disease outbreaks has been reported in the last decades [21–24] and one in which destruction of native rainforests has been particularly extensive over the same time-period [25–27]. However, and in spite of the public-health implications of a possible link between deforestation and Chagas disease risk, the responses of Amazonian *Attalea* palms and their resident *Rhodnius* populations to anthropogenic landscape disturbance remain largely uncharted (but see [14, 17, 18, 27]).

Aiming to address this gap, here we investigate the effects of anthropogenic landscape disturbance on eastern-Amazonian populations of *Attalea* palms and palm-dwelling *Rhodnius*. Importantly, we used field-sampling methods and statistical-modeling strategies that allowed us to explicitly account for the imperfect detection of both palms and bugs. Taken together, our findings suggest that deforestation may favor palm-dwelling populations of Chagas disease vectors in Amazonia, and hint at critical areas for future work including field research and disease prevention.

## Methods

### Study setting

Fieldwork took place in the municipality of Araguatins, on the right bank of the Araguaia River in northern Tocantins state, Brazil (S1 Fig), in October 2014, April 2015, and June 2015. The original vegetation cover of Araguatins primarily corresponds to eastern Amazonian broadleaf forests, with some patches of Cerrado savanna to the south and east [14]. The Instituto Brasileiro de Geografia e Estatística (IBGE) reports that by 2017 about one-third of the land had already been converted to cattle pasture (~88,500 ha, of which ~7600 were ungrazed), while natural forests covered some 17% of the land (~43,500 ha) [28]. We chose to work in Araguatins because invasion of houses by adult, palm-dwelling *Rhodnius* spp. is common in the municipality, with 1356 invasion events recorded by routine surveillance in 2010–2016 [14].

### Study localities, landscape classes, and sampling design

In consultation with municipal and state health officials, we selected two rural localities (which we will call simply “L1” and “L2” hereafter) located ~30 km apart (~50 km by road) and with similar settlement and land-use patterns–small house clusters plus scattered farms (yielding < 2.0 residents per km^2^) on land originally covered by moist broadleaf forest but now including three landscape classes clearly differing in their degree of anthropogenic disturbance [27]:

- Old-growth forest (“OGF” hereafter), i.e., relatively large patches (> 500 ha) of mature, well-preserved forest–either apparently uncut or in late-stage recovery;
- Cattle pasture (“CP” hereafter), with forest replaced by planted grasses that are maintained by periodic slash-and-burn of forest regrowth; and
- Young secondary forest (“YSF” hereafter) patches (> 200 ha) in the early stages of recovery after land converted to cattle pasture is abandoned.

In each locality, and based on field observations and inspection of satellite imagery, we selected survey areas representing each landscape class; we then established one linear transect in YSF and CP and three linear transects in the more heterogeneous OGF. In total, then, we worked in 5 transects × 2 localities = 10 transects: six in OGF, two in YSF, and two in CP (Table 1). Naturally-growing *Attalea* palms were present in all survey areas and transects. Transects were approximately straight; length was planned to be 2 km, but one OGF transect was only ~1.2-km long because of terrain difficulties. We therefore sampled across ~19.2 km overall (Table 1). We walked a central trail along each transect to sample palms and triatomines. For palms, we used a distance-sampling approach [29], and, for bugs within palms, a repeated-sampling approach [17, 18, 30, 31]. These approaches provided us with the information we needed to estimate palm and bug detection probabilities and, hence, correct presence and abundance (or density) estimates after accounting for detection failures; we now explain each sampling strategy in detail.

**Table 1:** *Attalea* palm distance sampling. Observed abundance and density (per hectare, ha) of adult *Attalea* palms along 10 linear transects in three landscape classes of two eastern-Amazonian localities.

Locality	Landscape	Transect			*Attalea* palms recorded	
		Code	Length (m)	Area (ha)	Number	Density (ha^−1^)
L1	Old-growth forest	OGF1	1200	9.6	140	14.58
	Old-growth forest	OGF2	2000	16.0	416	26.00
	Old-growth forest	OGF3	2000	16.0	251	15.69
	Overall OGF L1	-	5200	41.6	807	19.40
	Cattle pasture	CP1	2000	16.0	82	5.13
	Young secondary forest	YSF1^a^	2000	16.0	80	5.00
L2	Old-growth forest	OGF4	2000	16.0	174	10.88
	Old-growth forest	OGF5	2000	16.0	175	10.94
	Old-growth forest	OGF6	2000	16.0	195	12.19
	Overall OGF L2	-	6000	48.0	544	11.33
	Cattle pasture	CP2	2000	16.0	180	11.25
	Young secondary forest	YSF2^a^	2000	16.0	163	10.19
Both	Old-growth forest	-	11,200	89.6	1351	15.08
	Cattle pasture	-	4000	32.0	262	8.19
	Young secondary forest	-	4000	32.0	243	7.59
Total	All	All	19,200	153.6	1856	12.08

^a^Visual inspection of Landsat satellite imagery (https://earthexplorer.usgs.gov/) suggested that, at the time of sampling, secondary forest had been growing for ~4–5 y in L1 and for ~7–10 y in L2.

#### Palms: Distance sampling

**Fig 1:**
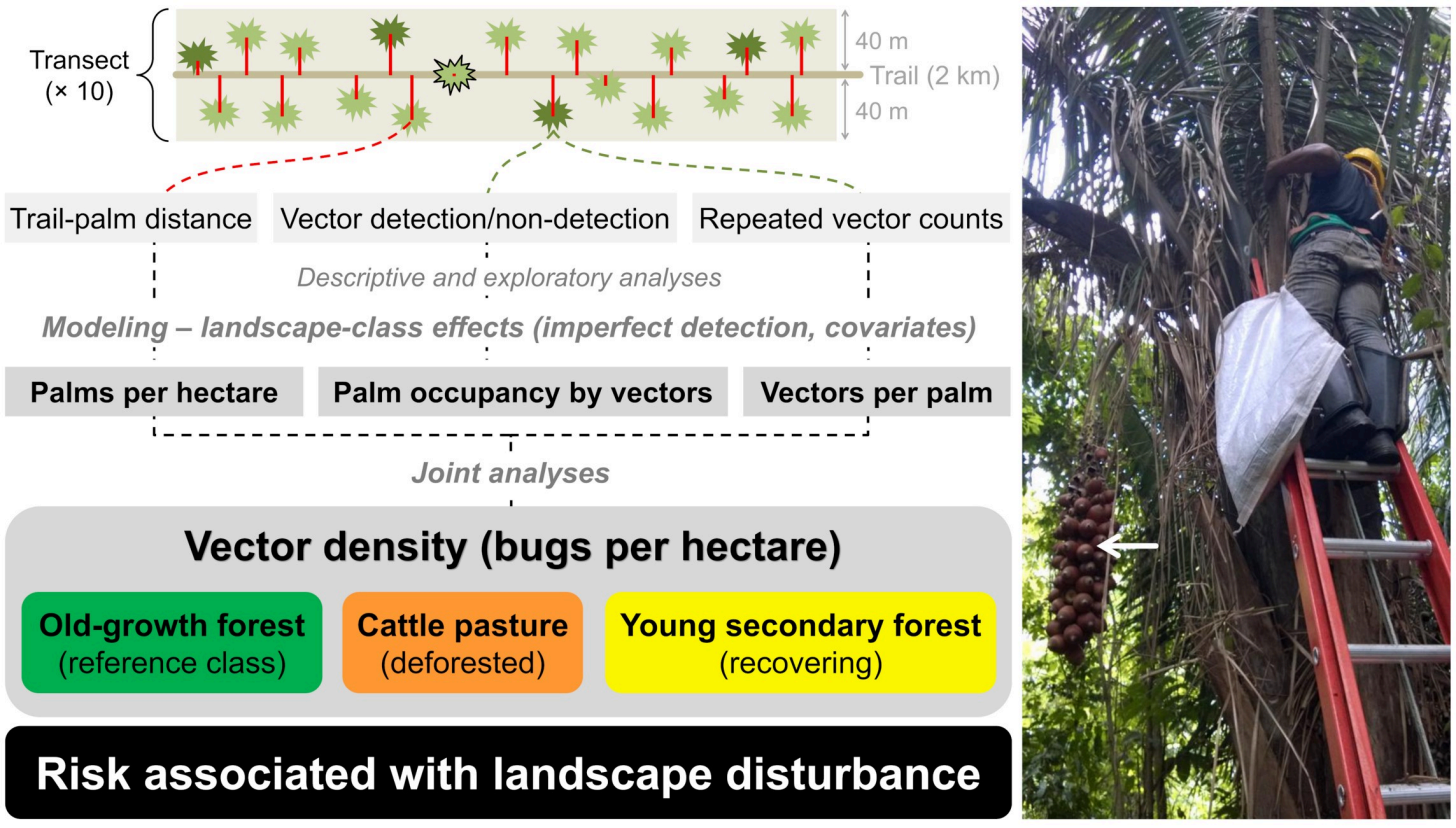
Field-sampling design, main data features, and data-analytical strategy. We sampled 10 transects by walking a central trail and measuring the perpendicular distance (red lines) between each adult *Attalea* palm seen (green star-like shapes) and the center of the trail. Palm detection was assumed certain for palms at zero distance (black-outlined palm). A systematic sample of evenly-spaced palms (darker green) was selected for triatomine-bug sampling in each transect. The right-hand-side picture illustrates bug searches, which included one direct catch on the palm plus a second, independent catch in palm debris collected in a bag; the white arrow points to a palm-fruit cluster. Palm-trail distances, bug detections/non-detections and repeated bug counts were used for descriptive/exploratory analyses and for inferential statistical modeling–in which we estimated covariate-adjusted landscape-disturbance effects after taking palm and bug detection failures into account. Joint analyses of palm and bug density allowed us to derive estimates of bug density per unit area in three landscape classes–old-growth forest, cattle pasture, and young secondary forest. We thus were able to make inferences about whether and how anthropogenic landscape disturbance is associated with three putative proxies for Chagas disease risk–palm density, palm infestation, and vector density.

One observer walked the central trail of each transect and recorded every adult (i.e., flowering/fruiting) *Attalea* palm he saw on the trail or to either side of it (Fig 1). Using a laser meter (DLE 40; Bosch, Gerlingen, Germany), the observer measured the perpendicular distance between the center of the trail and the stem of each palm (Fig 1). Given the 40-m reach of the laser meter, we recorded only palms within 80-m strips (40 m on either side of the trail; Fig 1); we then marked each individual palm with a metal plate bearing a unique number. Thus, sampling covered ~19,200 × 80 ≈ 1,536,000 m^2^ (~153.6 ha), in which we listed all adult *Attalea* palms detected and their perpendicular distances to the trails (Table 1). This distance-sampling approach relies on the assumption that palms located on the trail (at “zero distance”) are detected with probability 1.0 (or 100%); the probability then decreases as a function of distance from the observer [29, 32] (see Fig 1 and **Data analyses** below).

#### Bugs: Repeated sampling

Using a systematic-sampling frame, we selected 30 adult *Attalea* palms for bug sampling in each transect; in two transects, however, a few palms could not be sampled because of safety concerns, so the final sample comprised 280 instead of 300 palms. These palms were approximately evenly spaced (every ~50–60 m) along the transects (Fig 1), and were independently sampled for bugs by two observers. One observer climbed the palm using a ladder and manually collected bugs directly from the crown and upper stem during ~10 min (“direct catch”). Then, he gathered fibers, dead leaves, epiphytes, and other plant debris present on the palm and placed them in a large bag; this was done until the bag was filled or there was no more debris to collect, and the bag was then handed to a second observer. Unaware of the results of the direct catch, the second observer spread bag contents on a white canvas and searched them for bugs during 5 min (“bag catch”). The results of each direct catch and each bag catch were recorded separately; this yielded, for each palm in our sample, a “vector detection/non-detection history” (bugs detected, coded “1”; or not, coded “0”) and two “vector counts” (number of bugs caught by each method) (Fig 1). We identified the bugs (as *R*. *pictipes* and *R*. *robustus s*.*l*.) by their external morphology; however, nymphs of *R*. *robustus*, *R*. *neglectus*, and *R*. *marabaensis*, all of which occur in the study region [4, 12, 14], are either indistinguishable or yet to be described, and we therefore pooled all *Rhodnius* spp. for quantitative analyses (see below). Bugs were collected under license Sisbio 22404–2 issued by the Sistema de Autorização e Informação em Biodiversidade, Ministério do Meio Ambiente, Brazil.

### Data analyses

Our dependent variables were defined as follows: (i) for palm abundance/density analyses, the perpendicular distances between the center of the trail and the stem (at the height of the observer’s chest) of each palm seen from that trail (S1 Dataset); (ii) for the analyses of palm occupancy by triatomines, the history of bug detections and non-detections by each of two independent observers in each palm (S2 Dataset); and (iii) for bug density analyses, the counts of bugs seen by each of two independent observers in each palm (S2 Dataset). Anthropogenic landscape disturbance was the focal predictor in all our analyses; it was indexed by a three-level factor (OGF, CP, YSF) as described above, with OGF always used as the reference level. In addition, we wanted to control for the possible effects of some key potential confounders. These included (i) locality, for which we built a two-level factor, and, for palm infestation and bug density analyses, individual-palm measures of (ii) stem height and (iii) the semi-quantitative “organic score” of Abad-Franch et al. [33]. Stem height and organic score were standardized to mean zero and standard deviation (SD) one for modeling.

Our overall analytic strategy is depicted in Fig 1. We first described and summarized our data in tables and figures, and used generalized linear models (GLMs) fitted in R 3.6.3 [34] with the *glmmTMB* 1.0.1 [35] and *pscl* 1.5.2 [36] packages to explore associations between the dependent and independent variables described above. Inferential analyses were also run in R 3.6.3 [34] and comprised four main steps:

- First, we estimated *Attalea* palm abundance using distance-sampling data and models [29]. We used the *Distance* 1.0.1 package [32] to select the “key function” describing how detection probability decreases from *p* = 1.0 at zero distance (i.e., for palms located on the trail) to some lower value at the maximum distance (here, 40 m) (Fig 1). After exploratory analyses (see below) suggesting substantial variation of palm detection probabilities across localities and landscapes, we fitted separate landscape-class models for each locality. We tried the “half-normal” and “hazard-rate” key functions; Akaike information criterion (AIC) scores selected the half-normal as the best-fitting function [29, 32, 37]. Locality-specific and overall palm densities per hectare (ha) were then estimated for each landscape class. As for the rest of our main analyses, we focus on relative palm abundance/density across landscape classes, using OGF as the reference class.
- Second, we estimated palm occupancy (or infestation) by *Rhodnius* spp. in each landscape class using bug detection/non-detection data. To formally account for bug-detection failures, we used the single-season site-occupancy models of MacKenzie et al. [30, 38] implemented in *unmarked* 0.13–2 [39]. Again, we focused on estimating landscape-disturbance effects–i.e., how the odds of palm infestation varied in CP and YSF relative to the reference class, OGF. Our focal model estimates these landscape-class effects while controlling for variation between localities and among palms with different stem-height and organic-score values. We also fitted a simplified model without the locality confounder to estimate average landscape-disturbance effects on palm infestation.
- Next, we used the repeated-counts (or *N*-mixture) models of Royle [31] and *unmarked* 0.13–2 [39] to estimate the effects of landscape disturbance on vector density–with CP and YSF density estimates evaluated, as above, relative to those for OGF. These models use repeated counts and a mixture of two discrete distributions: (i) a binomial distribution to model vector detections/non-detections and (ii) a Poisson, zero-inflated Poisson, or negative-binomial distribution to model vector counts [39]; in our case, AIC selected the zero-inflated Poisson as the best-fitting distribution for the bug-count submodel. We again used a model with landscape class as the focal predictor and with locality, palm stem height, and palm organic score as covariates; similar to palm infestation analyses, we also fitted a simplified model without locality effects.
- Finally, we used our density estimates (i.e., palms per ha and bugs per palm) across landscape classes to derive a measure of relative bug density per unit area–that is, a measure of how bug population sizes change in disturbed landscapes (CP and YSF) relative to better-preserved forests (OGF) (Fig 1).

Implicit in these analyses is the notion that palm density, palm infestation, and bug density can be viewed as putative proxies for Chagas disease risk (see, e.g., [7–13, 16–19, 21, 22, 33]).

### Bug infection with *Trypanosoma cruzi*

We also aimed at estimating *T*. *cruzi* bug-infection frequency across landscape classes as a more proximal measure of disease risk. To do this, we ran duplicate quantitative PCR (qPCR) assays targeting the repetitive region of the *T*. *cruzi* nuclear satellite DNA (following [40]; see details in S1 Text) on DNA extracted from the abdomens of (i) the bugs caught during the field activities described above and (ii) 11 additional bugs (not included in bug presence/density analyses) caught in a separate field trip to the same transects. Replicate qPCRs yielded a *T*. *cruzi* detection/non-detection history for each bug, and we used single-season site-occupancy models [30, 38, 39] to estimate qPCR sensitivity and bug-infection frequency with *unmarked* 0.13–2 [39]. Bug-infection data, however, turned out to be too sparse to confidently model landscape-disturbance effects, and we therefore will mention these results only briefly; full details on methods and findings are provided in S1 Text.

## Results

### Descriptive results and exploratory analyses

**Fig 2:**
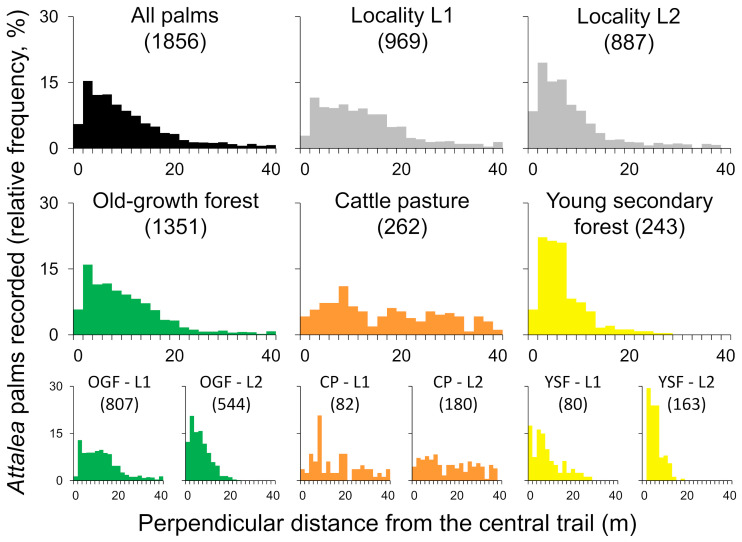
*Attalea* palm detections. The histograms show the relative frequencies of adult *Attalea*-palm detections (vertical axes, all scaled to a 30% maximum) as a function of perpendicular distance (horizontal axes; 0–40 m) from linear trails established in 10 transects representing three landscape classes in two eastern-Amazonian localities (“L1” and “L2”). The upper row shows overall and locality-specific results. Landscape class-specific results (middle row) show how detections clustered at shorter distances in young secondary forest (“YSF”), but were spread over all distances in deforested cattle pasture (“CP”); the pattern was somewhat intermediate for old-growth forest (“OGF”). The lower row shows results stratified by landscape class and locality. The number of palm detections used to build each graph is given in parentheses.

Table 1 summarizes the results of distance sampling; overall, we recorded 1856 adult *Attalea* palms (1489 *A*. *speciosa*, 367 *A*. *maripa*) along our 10 transects. As expected, the number of palm detections declined as perpendicular distance to the trails increased (Fig 2). The number of palm detections appeared to fall more steeply with distance in YSF than in the open CP transects, with a somewhat intermediate decline in OGF (Fig 2). More detailed graphs suggested that, for each landscape class, the rate at which palm detections decreased with distance varied also between the two study localities (Fig 2, lower row); this led us to model landscape class-specific palm-detection functions separately for localities L1 and L2 (see **Inferential analyses: modeling** below).

We detected infestation by *Rhodnius* spp. in 18 of the 280 palms systematically sampled along our 10 transects; these 280 palms had a mean stem height of 4.75 m (SD 1.4; range, 1.50–8.33) and a mean organic score of 0.73 units (SD 0.8; range, 0.25–3.00) (Table 2, S2 Dataset). An intercept-only GLM (binomial distribution, logit link function) quantified observed palm infestation at 6.43% (95% confidence interval [CI] 4.09–9.97%). Exploratory GLMs suggested that infestation odds varied little with palm organic-score values; moderately between localities and with palm stem height; and substantially among landscape classes–with palm infestation detected more frequently in disturbed landscapes, especially in CP but also in YSF, than in OGF (see S1 Table).

**Table 2:** Sampling for *Rhodnius* spp. in 280 adult *Attalea* palms: Descriptive results.

Locality	Landscape	Palms sampled	Palms infested^a^	Bugs caught
L1	Old-growth forest^b^	84	0	0
	Cattle pasture	16	11	23
	Young secondary forest^c^	30	2	3
L2	Old-growth forest^b^	90	2	4
	Cattle pasture	30	2	2
	Young secondary forest^c^	30	1	1
Both	Old-growth forest	174	2	4
	Cattle pasture	46	13	25
	Young secondary forest	60	3	4
Total	All	280	18	33^d^

^a^Number of palms in which we detected at least one triatomine bug (here, *Rhodnius* spp.).

^b^Note that three old-growth forest transects (but just one cattle pasture and one young secondary forest) were sampled in each locality.

^c^Visual inspection of Landsat satellite imagery (https://earthexplorer.usgs.gov/) suggested that, at the time of sampling, secondary forest had been growing for ~4–5 y in L1 and for ~7–10 y in L2.

^d^Eleven additional bugs caught in a separate field trip to the same transects were used for *Trypanosoma cruzi* detection assays, but were not included in our analyses of palm infestation and bug density.

The productivity of our bug-catch efforts was overall low. On a per-palm basis, bug catches were somewhat lower in OGF (0.023 bugs per palm) than in YSF (0.067), and much higher in CP (0.544 bugs per palm) (Table 2). Exploratory zero-inflated Poisson GLMs (log link) again suggested that the main source of variation was the degree of landscape disturbance, with bug densities much higher in CP than in OGF and a smaller, more uncertain difference between YSF and OGF (see S2 Table).

Duplicate qPCR assays detected *T*. *cruzi* infection in 5 bugs out of 44 (including 11 caught in a separate field trip; 11.4%). Infected bugs were all nymphs (stages NI to NIII), each caught in a different palm–four in CP and one in YSF. None of the four bugs caught in two OGF palms yielded *T*. *cruzi* detections (see details in S1 Text).

### Inferential analyses: Modeling

#### *Attalea* palm abundance/density

**Fig 3:**
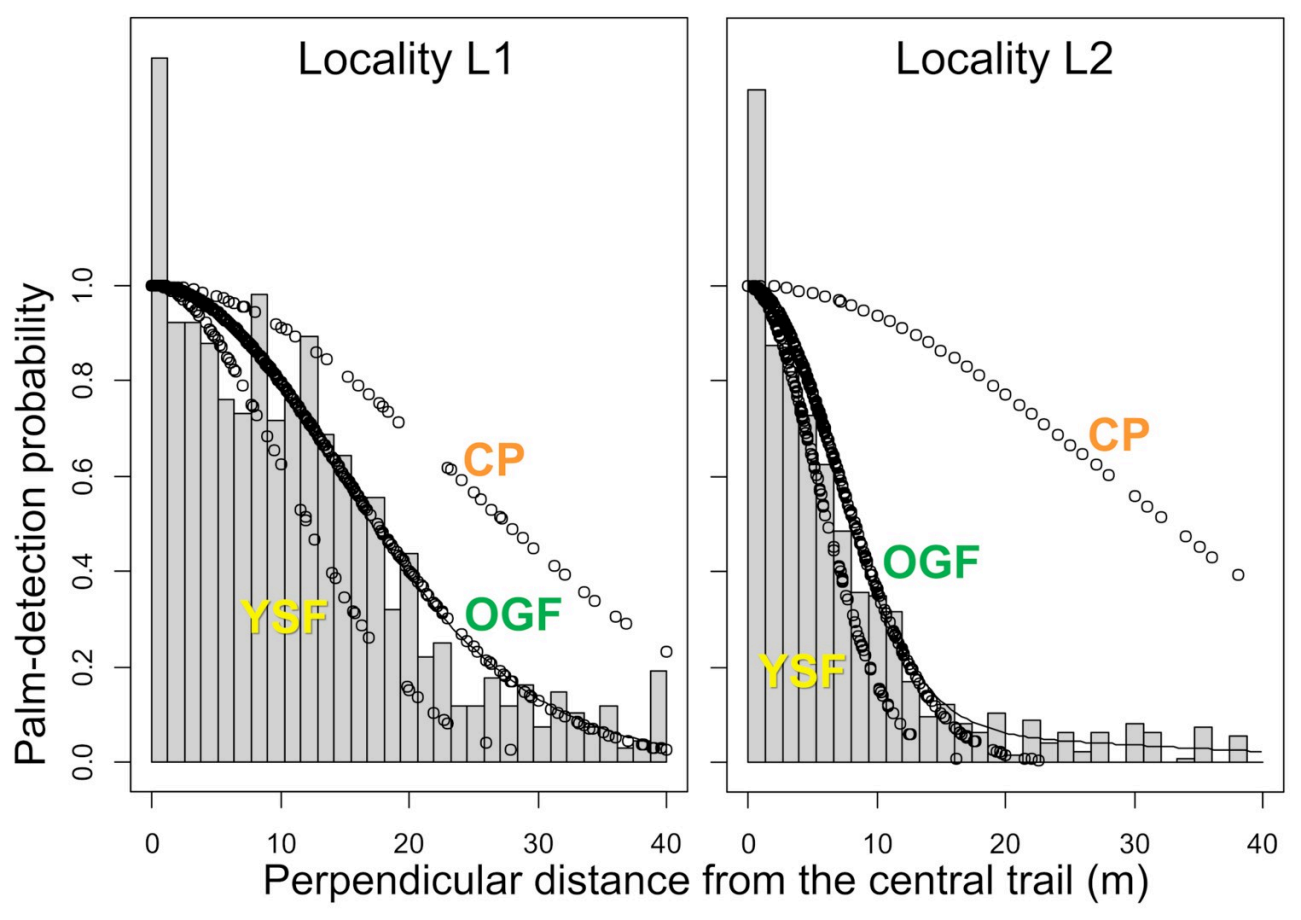
Estimated decline of *Attalea* palm detection probabilities as a function of distance. Results are shown by locality (“L1” and “L2”) and by landscape class (“OGF”, old growth forest; “CP”, cattle pasture; “YSF”, young secondary forest) within localities. Gray bars are overall palm-detection frequencies.

The rate of decline of palm-detection probabilities with increasing distance was faster in the more densely vegetated transects, and particularly in YSF, than in deforested CP (Fig 3). Thus, estimates of the detection function scale parameter (i.e., the SD of the half-normal function) were consistently smaller in YSF than in OGF–and the largest estimates were for CP transects (Table 3). After these differences in palm-detection probabilities were accounted for, our analyses suggest that the density of *Attalea* palms declined sharply in CP relative to OGF, with the former retaining only about 20–30% of the adult palms present in preserved forest patches of the same localities. Recovery of adult palm populations was apparent in our YSF transects, with density estimated as ~60% lower than in OGF in locality L1, where YSF was ~4–5 years old, but as ~20% higher in locality L2, where YSF was ~7–10 years old (Table 3). Thus, our analyses suggest that most adult *Attalea* palms are felled during clearcut for cattle ranching, but also that palm populations recover fairly quickly as deforested land is abandoned and secondary forest grows again.

**Table 3:** Palm distance-sampling modeling results. *Attalea* palm detection (scale parameter of the half-normal function) and palm-density estimates across landscape-disturbance classes.

Locality	Landscape	Palm detection (scale parameter)			Palm density (per hectare)				
		Estimate	SE	Meters^a^	Estimate	SE	Ratio	95% CI	
L1	OGF	2.70	0.15	14.83	42.05	8.34	Ref.	-	-
	CP	3.15	0.14	23.41	7.66	0.86	0.18	0.10	0.32
	YSF^b^	2.33	0.16	10.31	15.49	1.31	0.37	0.19	0.70
L2	OGF	1.95	0.14	7.02	51.53	2.41	Ref.	-	-
	CP	3.32	0.14	27.77	15.21	1.27	0.30	0.28	0.31
	YSF^b^	1.66	0.14	5.28	61.62	3.72	1.20	1.19	1.21

OGF, old-growth forest; CP, cattle pasture; YSF, young secondary forest; SE, standard error; CI, confidence interval; Ref., reference class (palm density taken to be 1.0).

^a^Exponentiated scale parameter; the lower the scale parameter value, the faster the decline of detection probabilities with increasing distance between palms and the transect’s central trail.

^b^Visual inspection of Landsat satellite imagery (https://earthexplorer.usgs.gov/) suggested that, at the time of sampling, secondary forest had been growing for ~4–5 y in L1 and for ~7–10 y in L2.

#### *Attalea* palm occupancy by *Rhodnius* spp

**Fig 4:**
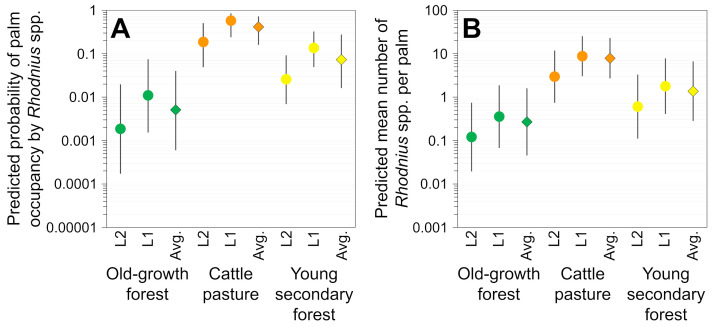
Effects of deforestation on adult *Attalea* palm occupancy (or infestation) by *Rhodnius* spp. **(A)** and on the number of *Rhodnius* spp. per palm **(B)**. The graphs show model-predicted probabilities of palm occupancy (**A**) and model-predicted mean number of bugs per palm (**B**) for three landscape classes in two localities (“L1” and “L2”) of eastern Amazonia. “Avg.” values (diamonds) are average predictions derived from simplified models without locality effects. All predictions are for “typical” palms with mean stem height (4.75 m) and mean organic score (0.73 units); note that the *y*-axes are on log10 scale. Error bars are 95% confidence intervals.

Our focal site-occupancy model suggests that there was substantial variation in palm infestation odds across landscape classes, localities, and palm traits. Table 4 shows the adjusted effects estimated by this model, and Fig 4A illustrates model predictions for each landscape class and locality. A simplified model without locality effects suggests that bugs were present, on average, in just about 0.51% (CI 0.06–4.02%) of “typical” palms (i.e., with stem height and organic score at the overall means of 4.75 m and 0.75 units, respectively) located in OGF, *vs*. 41.50% (CI 15.97–72.55%) of similar palms in CP and 7.32% (CI 1.61–27.65%) in YSF (Fig 4A). Thus, relative to OGF, infestation estimates for the “typical” palm were, on average, ~80.9 times higher in heavily disturbed CP landscapes and ~14.3 times higher in recovering YSF (Fig 4A). Both the focal model and the simplified model fitted the data much better, as judged by large AIC differences (> 40 units), than a “null” model estimating only intercepts.

**Table 4:** Adjusted effects (logit scale) of anthropogenic landscape disturbance on adult *Attalea* palm occupancy by *Rhodnius* spp.

Term	Estimate (SE)	CI lower	CI upper
Intercept	−6.286 (1.215)	−8.666	−3.905
Landscape disturbance			
Old-growth forest	Reference class	-	-
Cattle pasture	4.816 (1.141)	2.579	7.053
Young secondary forest	2.655 (1.100)	0.499	4.811
Confounders			
Locality			
L2	Reference class	-	-
L1	1.785 (0.821)	0.176	3.394
Palm traits			
Organic score (0.8-unit increase)^a^	0.824 (0.369)	0.101	1.546
Stem height (1.4-m increase)^a^	1.297 (0.496)	0.325	2.268

SE, standard error; CI lower and CI upper, lower and upper limits of the 95% confidence interval.

^a^Variables standardized to mean 0.0 and standard deviation (SD) 1.0; effect estimates therefore correspond to an increase of 1 SD in the covariate value (indicated in parentheses).

#### Density of *Rhodnius* spp. colonies in *Attalea* palm crowns

In line with the occupancy analyses above, our focal *N*-mixture model estimates strong positive effects of anthropogenic landscape disturbance on the mean density of *Attalea* palm-dwelling *Rhodnius* spp. The results are summarized in Table 5 and Fig 4B. This *N*-mixture model predicts higher mean bug densities in our CP palms (~5.48±0.98 SE bugs per palm; median = 2.20, inter-quartile range [IQR] 1.25–8.35) than in OGF palms (~0.26±0.03 SE; median = 0.15, IQR 0.09–0.26), with intermediate expectations for palms sampled in YSF (~0.79±0.11 SE; median = 0.43, IQR 0.23–0.97 bugs per palm). A simplified model without locality effects suggests that individual OGF palms (of mean stem height and mean organic score) harbored just about 0.27 (CI 0.05–1.60) bugs on average, *vs*. 8.78 (CI 3.04–25.34) for CP palms and 1.37 (CI 0.28–6.64) for YSF palms (Fig 4B). Thus, relative to OGF, bug-density estimates for the “typical” palm were, on average, ~29.3 times higher in heavily disturbed CP landscapes and ~5.1 times higher in recovering YSF (Fig 4B). AIC scores were > 30 units smaller for the focal and simplified models than for a “null”, intercepts-only model.

**Table 5:** Adjusted effects (log scale) of anthropogenic landscape disturbance on the density of *Rhodnius* spp. in adult *Attalea* palms.

Term	Estimate (SE)	CI lower	CI upper
Intercept	−2.115 (0.930)	−3.938	−0.293
Landscape disturbance			
Old-growth forest	Reference class	-	-
Cattle pasture	3.203 (0.618)	1.991	4.414
Young secondary forest	1.613 (0.755)	0.134	3.091
Confounders			
Locality			
L2	Reference class	-	-
L1	1.085 (0.502)	0.100	2.070
Palm traits			
Organic score (0.8-unit increase)^a^	0.677 (0.213)	0.259	1.095
Stem height (1.4-m increase)^a^	0.534 (0.534)	0.061	1.008

SE, standard error; CI lower and CI upper, lower and upper limits of the 95% confidence interval.

^a^Variables standardized to mean 0.0 and standard deviation (SD) 1.0; effect estimates therefore correspond to an increase of 1 SD in the covariate value (indicated in parentheses).

#### Infection of *Attalea*-dwelling *Rhodnius* spp. with *Trypanosoma cruzi*

As noted above, our bug-infection data were too sparse to allow modeling heterogeneity across landscape-disturbance classes. A simple, intercepts-only site-occupancy model estimates mean bug-infection frequency at Ψ = 0.118 (CI 0.050–0.256), and mean qPCR sensitivity at *p* = 0.80 (CI 0.50–0.94) for each qPCR assay.

### Joint analyses: Vector density per unit area

Table 6 shows a summary of our estimates of adult *Attalea* palm density (from the distance-sampling models in Table 3) and of the mean predicted number of *Rhodnius* spp. per palm (from the *N*-mixture model in Table 5) across landscape classes. Using those estimates, we derived an approximation to the expected number of *Rhodnius* spp. per hectare in each landscape class (Table 6). The results suggest that, in our two study sites of eastern Amazonia, disturbed landscapes supported from ~2.5 (YSF; approximate CI 1.7–3.2) to ~5.1 (CP; approximate CI 3.3–6.9) times as many palm-dwelling *Rhodnius* per unit area as did better-preserved OGFs.

**Table 6:** Estimates of adult *Attalea* palm density, mean number of *Rhodnius* spp. per palm, and expected *Rhodnius* spp. per hectare (ha) across three landscape classes in two localities of eastern Amazonia.

Landscape class	Palms/ha	Bugs/palm^a^	CI^b^		Bugs/ha^c^	CI	
Estimates							
Old-growth forest	47.13	0.26 (0.03)	0.20	0.33	12.37	9.22^d^	15.53^d^
Cattle pasture	11.44	5.48 (0.98)	3.52	7.44	62.65	40.24^d^	85.06^d^
Young secondary forest	38.56	0.79 (0.11)	0.56	1.01	30.26	21.46^d^	39.07^d^
Ratios							
Old-growth forest (ref.)	1	1	-	-	1	-	-
Cattle pasture	0.24	20.87	13.54^e^	28.62^e^	5.06	3.25^f^	6.87^f^
Young secondary forest	0.82	2.99	2.15^e^	3.89^e^	2.45	1.73^f^	3.16^f^

^a^Mean (standard error) of palm-specific predictions from the *N*-mixture model of Table 5.

^b^Approximate confidence interval calculated as the mean ± 2 standard errors [e.g., disregarding rounding errors, 0.20 = 0.26 − (2 × 0.03), and 0.33 = 0.26 + (2 × 0.03)].

^c^Computed as the product of palm density per hectare (second column) and bug density per palm (third column) (e.g., disregarding rounding error, 12.37 = 47.13 × 0.26).

^d^Approximate confidence interval computed as the product of palm density per hectare and the lower and upper limits of the bug-density CI (e.g., disregarding rounding error, 9.22 = 47.13 × 0.20).

^e^Approximate confidence interval computed as the ratio of each CI limit and the mean per-palm bug-density estimate for old-growth forest (e.g., disregarding rounding error, 13.54 = 3.52/0.26).

^f^Approximate confidence interval computed as the ratio of each CI limit and the mean bug-density estimate for old-growth forest (e.g., disregarding rounding error, 3.25 = 40.24/12.37).

## Discussion

Whether and how anthropogenic landscape disturbance affects human infectious-disease risk is a debated topic with important implications for both disease prevention and biodiversity conservation [41–45]. The links between landscape disturbance and disease risk, however, can be complex and elusive, particularly for multi-host, vector-borne pathogens like *T*. *cruzi* [41, 44–50]. In this study, we provide evidence suggesting that anthropogenic landscape disturbance and deforestation may favor Chagas disease vector populations infesting palms of eastern Amazonia. Using field-sampling methods and statistical-modeling approaches that allowed us to formally account for detection failures, we found that landscape disturbance driven by cattle ranching was associated with an overall decline of adult *Attalea* palm densities, but also with clearly increased odds of *Attalea* palm infestation by *Rhodnius* spp. and with substantially higher *Rhodnius* population densities. Taken together, our results suggest that eastern-Amazonian deforested landscapes may support between 2.5 and 5.1 times more *Attalea*-dwelling *Rhodnius* spp. per unit area than better-preserved old-growth forests.

### Palms, bugs, and anthropogenic landscape disturbance in eastern Amazonia

**Fig 5:**
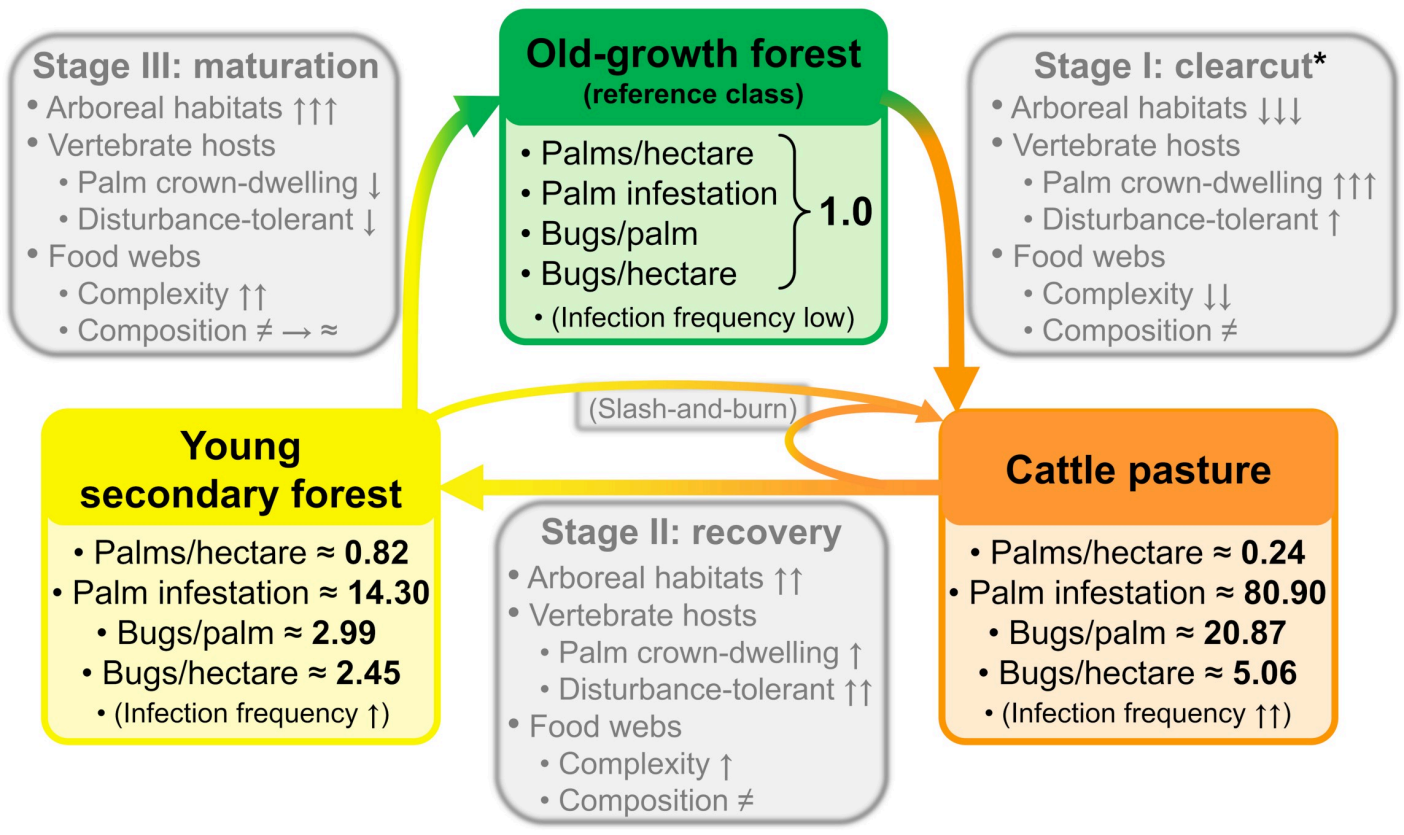
Effects of anthropogenic landscape disturbance on *Attalea* and *Rhodnius* spp.: A summary of results and hypotheses. The original old-growth forest (green box) is our “baseline” or “reference class”, and has therefore 1.0 values for all putative risk proxies (palms per hectare, palm infestation, and bug density per palm and per hectare; we could not quantify disturbance effects on vector-infection frequency). The right-hand-side grey box (“Stage I”) summarizes changes associated with transformation of old-growth forest into cattle pasture; note that some palms are usually spared during clearcut (hence the asterisk). The orange box summarizes changes, relative to old-growth forest, of our putative risk proxies. Cattle pasture is maintained through slash-and-burn; when abandoned, it recovers (“Stage II” box) into secondary forest. The yellow box again summarizes changes, relative to old-growth forest, of our putative risk proxies. If left undisturbed, secondary forests mature (“Stage III” box) into old-growth forests and the cycle is eventually completed. Note that the grey boxes show hypothetical processes (involving availability of arboreal habitats, vertebrate-host responses, and food-web features) that we suggest should be the focus of future research; the green, orange, and yellow boxes summarize the main results (presented as ratios–relative to old-growth forest) of the present study. See S1 Text for details.

The process of anthropogenic landscape disturbance in Amazonia typically starts when mature forests are felled and the land transformed into cattle pasture [27]. Large palms such as arborescent *Attalea* may be spared during clearcut because they provide shade for the livestock and because their fruits, seeds, and fronds are used for many purposes [27]. Pastures are usually maintained through slash-and-burn of forest regrowth; when they are abandoned, secondary forest recovers–and, if left undisturbed, matures back into old-growth forest [51]. Our three landscape classes were selected to roughly represent the main stages of this “cycle” of disturbance, recovery, and maturation (Fig 5). Below we discuss, based on our findings, how adult *Attalea* and their associated *Rhodnius* populations may respond to each stage of the cycle; we, in addition, outline a series of hypotheses about what major processes may underpin the patterns we discovered, and suggest future research directions.

#### Stage I: Clearcut

Our findings indicate that deforestation for cattle ranching has a negative impact on adult *Attalea* populations; the intuition (or perception) that palms are more abundant in CP than in preserved landscapes [17, 27, 33, 52] seems to be driven by the fact that palms are just easier to see in open land than in denser forest (Table 3, Fig 3). Our results suggest that as many as ~70–80% of adult *Attalea* palms may be felled as OGF is converted to CP. However, since almost all trees and undergrowth are also felled, the few uncut palms may become the most abundant (or even only) arboreal habitats available in CP [27]. Disturbance-tolerant arboreal vertebrates might therefore end up using palm crowns more often in CP than in well-preserved OGF. If this leads to a higher and steadier availability of vertebrate hosts, then bug colonies in CP palms could be less prone to local (palm-level) extinction and could grow denser than those in OGF. Bug infections might also be more frequent in CP than in OGF if, in addition, disturbance-tolerant vertebrates were competent *T*. *cruzi* reservoirs–with, e.g., opossums and some rodents having both characteristics [50]. We suggest that this process may underpin the observed patterns of higher infestation odds, higher bug densities, and likely more frequent bug infection with *T*. *cruzi*, in our CP palms (Fig 5). This hypothesis has some support from cross-sectional observations (e.g., [7, 47, 53, 54]), but needs to be more thoroughly evaluated. A suitable design would select study sites that represent OGF and CP and then combine (i) experimental manipulation of access to palm-crowns by non-flying vertebrates (e.g., by fitting metal bands around the stems of solitary palms [55]) with (ii) a survey of the vertebrate fauna available to the bugs (by direct sampling or through bug-bloodmeal analyses [47, 56, 57]); we suggest that (iii) modeling detection failures (of bugs, parasites, and hosts) would provide more reliable estimates of population parameters and associated uncertainties [30]. We also note that ecological release from predators and pathogens in simplified (or otherwise modified) food webs could also play a role in favoring bug populations in palms of heavily disturbed landscapes (Fig 5). For example, some bug predators might become rarer in CP palms, and a drier palm-crown microclimate might protect bugs from fungal pathogens [7, 47]. Again, however, these hypotheses have yet to be tested in the field–in part, perhaps, because adequately sampling the complex food webs of large palm-crowns can be very difficult [58]. Multi-species hierarchical models [59, 60] and DNA metabarcoding [56, 57, 61–63] could help us understand how palm-crown communities respond to landscape disturbance–and how this affects vector and parasite populations.

#### Stage II: Recovery

*Attalea* palms are among the pioneers of secondary-forest regrowth when Amazonian land converted to CP is abandoned [27]. Our data reflect this as an increase of palm density estimates in YSF relative to CP; the much higher YSF palm density in L2 (~62 adult *Attalea* per ha) than in L1 (~16 per ha) is likely due to a longer period of forest regrowth in our L2 transect–as also suggested by the faster rate of decline of palm detections (Fig 3) and by the taller stems of YSF palms in L2 (mean 4.7 m, SD 1.5) than in L1 (3.9 m, SD 0.9) (see S2 Dataset). Landsat images indeed suggested that, at the time of sampling, YSF had been growing for about 7–10 years in our L2 transect but only for about 4–5 years in our L1 transect (see Tables 1–3). As adult palms are recruited in growing YSF, palm infestation by *Rhodnius* spp. and bug density both seem to start declining (Figs 4 and 5). This suggests that at least some of the factors possibly favoring bug infestation and density after clearcut may progressively shift back towards their original states (Fig 5). Thus, (i) increased availability of arboreal microhabitats including palms might reduce the intensity of palm-crown use by vertebrates; (ii) some disturbance-sensitive vertebrates (including less competent *T*. *cruzi* reservoirs) might return to recovering secondary forest; and (iii) palm-crown communities and microclimates might gradually become more similar to the ones in OGF (Fig 5). Hypothetically, the combined effects of these mechanisms would put bug populations back on a progressively tighter “ecological control”, with declines of both palm infestation and bug density (Fig 5). The somewhat lower infestation and bug density we found in the older, higher palm-density YSF transect of L2 (Table 3, Fig 4) might reflect these dynamics. Sampling across secondary forests of different ages but otherwise similar, ideally within the general design outlined in the previous section, would provide the data needed to test this hypothesis.

#### Stage III: Maturation

As secondary forest mature, the populations of adult *Attalea* palms and woody trees recover, providing increasingly abundant habitat for arboreal vertebrates. Each individual palm-crown may thus become even less likely, on average, to be stably occupied by vertebrates, for which many alternative habitats would now be available. At the same time, palm-crown food webs might gradually regain complexity, including more diverse bug hosts, predators, pathogens, and competitors (Fig 5). Combined, reduced host availability and a tighter “ecological control” might result in bug colonies being smaller on average, and hence more prone (also on average) to palm-level local extinction (Fig 5). The pattern emerging from these dynamics would then be one of low palm infestation frequencies and low mean bug density–just as we observed in our OGF transects (Table 3, Figs 4 and 5). These results, combined, suggest that OGF mean bug-density figures are largely driven by fairly crowded colonies dwelling in a few palms. This is consistent with the common observation of clustering (or statistical overdispersion) in wild *Rhodnius* populations: especially in forested sites, most available palms are vacant, a few harbor small colonies, and a very small minority of (presumably higher-quality) palms support larger colonies [7, 17, 18, 33]. Finally, mature OGF likely supports an increasingly diverse vertebrate community; this might eventually result in the bugs feeding more frequently on incompetent or poorly-competent *T*. *cruzi* reservoirs, which would lead to lower bug-infection prevalence and, therefore, less intense circulation of the parasite (Fig 5). This “dilution-effect hypothesis” [41–44, 48] is particularly appealing for wild *T*. *cruzi* cycles such as those maintained by *Rhodnius* spp. in Amazonian palm crowns–but, again, is yet to be thoroughly tested.

### Caveats and strengths

There are a few caveats that readers should keep in mind when interpreting our findings. First, our bug data are very sparse. In particular, we found bugs in just 18 palms and counted just 33 bugs. This low bug yield was, based on our field experience, somewhat unexpected. Apart from the possibility of true lower palm infestation and bug density in our study area than elsewhere in Amazonia, low bug yield may also reflect our unbiased selection of palms via systematic sampling–in contrast with convenience sampling, in which palms suspected of harboring bug colonies (e.g., taller, larger-crowned palms) may be preferentially selected, whether consciously or unconsciously [18]. To check this possibility, we compared our 280 systematically-sampled palms with 252 “haphazardly selected” palms of the same two species (*A*. *speciosa* and *A*. *maripa*) studied by us in central-northern Amazonia (see [17] and S3 Dataset). A series of GLMs (S3 Table) shows that, on average, the palms in our current sample (i) were shorter, (ii) had lower organic-score values, (iii) yielded less bug detections, and (iv) yielded lower bug catches than the palms in [17]. The systematic sample selected for the present study, therefore, appears to be less biased towards tall, high-organic score palms; because both palm traits have a positive effect on infestation (Table 4; [17, 18]) and bug density (Table 5), our unbiased sample likely provides particularly realistic estimates of overall infestation and bug density in adult *A*. *speciosa* and *A*. *maripa*. In any case, we underscore that the sparseness of our data (i) did not lead to model convergence issues and (ii) is reflected in the large uncertainty associated with some of the effect estimates we report. In particular, the wide CIs of the landscape-class effect estimates reported in Tables 4 and 5 are a consequence of the very few bug detections in OGF. We therefore suggest that the key take-home message is that palm infestation was more frequent, and bug density higher, in heavily disturbed than in well-preserved landscapes, with a more precise quantification of these effects requiring further research. Another negative consequence of data sparseness was that we were unable to confidently model bug-detection probabilities as a function of covariates; instead, we had to assume that detection probabilities did not vary, e.g., across landscape classes or with palm traits. A better understanding of the bug-detection process may sharpen our views about vector population ecology and can help us strengthen vector control-surveillance [17, 64, 65]; we believe that this is an important topic for future research. Finally, with our limited sample we were also unable to quantitatively assess whether bug-infection frequency varied across landscape classes–all we can say is that we detected *T*. *cruzi* infection in five of 40 bugs caught in disturbed landscapes, but in none of the four bugs caught in OGF. However limited, these results show that the parasite was circulating in at least 31.3% of the 16 disturbed-landscape palms in which we detected infestation (Table 1 and S1 Text).

A second caveat is that we do not consider possible seasonal effects associated, e.g., with rainfall variation between drier (October, June) and rainier (April) sampling months. To our knowledge, however, earlier studies examining this possibility in *Attalea* palm-crown habitats have all reported negligible season effects on *Rhodnius* spp. occurrence or abundance [53, 66–68]. This is probably due, at least in part, to the stable, buffered microclimate typical of the large and structurally complex crowns of *Attalea* species such as *A*. *speciosa* or *A*. *butyracea* [53, 67]; in the smaller-crowned *Acrocomia*, both infestation and bug density may vary seasonally [69, 70].

Third, we do not have precise ground data on either the time elapsed since forest clearance or the intensity of disturbance in our study transects; instead, and in line with our previous work on the same topic (e.g., [17, 18]), we defined three landscape classes that, as mentioned in the **Methods** section, clearly differed in their degree of anthropogenic disturbance (see also [27, 51, 71]). While this (admittedly rough) approximation allowed us to test our main hypotheses about deforestation effects on palms and bugs, we believe that future studies should characterize disturbance age and intensity in more detail. As illustrated above for our two YSF transects, readily available satellite imagery can help estimate the age of secondary forests.

Finally, a general caveat of our report is that we have no measurement of human infection with *T*. *cruzi* in the study areas. Instead, and also in line with many prior studies [7–13, 16–19, 21, 22, 33, 47, 52, 53, 58, 66–73], we regard palm density, palm infestation, vector density, and (even if with more limited data) vector-infection frequency as putative proxies for Chagas disease risk.

In addition to a field-sampling plan designed to minimize palm-selection biases, the two main strengths of our study are (i) the explicit and formal treatment of detection failures (of palms, bugs, and parasites) and (ii) the fact that we probed the effects of landscape disturbance on three important components of Chagas disease risk–the vectors’ primary ecotopes, the vectors themselves, and (admittedly with limited results) the parasites those vectors carry. Previous similar studies taking imperfect detection into account addressed landscape-disturbance effects on palm occupancy by the bugs, but not on the palms themselves or on bug density or infection [17, 18]. Conversely, previous studies of landscape-disturbance effects on palm infestation, bug density, and bug infection (e.g., [7–9, 16, 33, 53, 58, 66–70, 72, 73]) did not account for detection failures, thereby assuming that bugs and parasites are both detected with probability 1.0 –a strong, overall unrealistic assumption [17, 18, 40]. As far as we are aware, imperfect detection of palms (or any other known triatomine-bug ecotope) has not been formally considered previously, including in studies of *Attalea*-palm responses to land-use change (e.g., [27, 71]).

## Conclusions and outlook

Our findings suggest that in rural eastern Amazonia anthropogenic landscape disturbance is associated with higher odds of palm-crown infestation by Chagas disease vectors and with higher vector-population densities–both per palm and per unit area. Vector infection with *T*. *cruzi* might also be more common in disturbed than in preserved landscapes. Taken together, these findings lend support to the view that wild *T*. *cruzi* vectors and humans widely coexist across the expanding disturbed landscapes of Amazonia [21]. As they disperse from palms, adult bugs often invade houses or other premises and may infect people directly or via food contamination [14, 15, 21–24, 52, 58, 72, 73]. This is currently the main mechanism underlying *T*. *cruzi* transmission to humans in Amazonia [21–24, 58], and an emerging one in other regions of the Americas where wild, native triatomines are important vectors of the parasite [5, 6].

Our results also suggest some directions for future work. First, understanding how palm-crown communities respond to deforestation would provide insight into the mechanisms by which landscape disturbance can affect Chagas disease risk [44]. Second, our study further highlights the need to quantify the human-health costs of deforestation, which may be instrumental for environmental-conservation advocacy [74, 75]. Finally, our findings suggest that incorporating measures of anthropogenic landscape disturbance into the spatial stratification of transmission risk could help enhance Chagas disease surveillance in Amazonia. The identification of higher-risk areas would allow for targeted action–including early case detection, integral patient care, or the promotion of safer food-management practices [76]. High-risk areas would also provide the ideal setting for well-powered trials of interventions aimed at reducing human-vector contact; this might include physically protecting homes and food-processing equipment with insect screens or managing peridomestic palms to lower organic-score values–thus reducing palm infestation odds and bug densities [17, 77].

The results we have presented, in sum, shed light on some important components of the long-hypothesized, yet still little-understood, link between deforestation and Chagas disease [78]. Further work is needed, however, to (i) better quantify landscape-disturbance effects on palm infestation, vector density, and vector infection; (ii) see if the response patterns we report also arise in other regions; and (iii) elucidate the mechanisms that underlie those patterns. Our methodological approach and main findings, at any rate, open new perspectives for research and preventive action; we believe that they are likely to be relevant for Amazonia as a whole and for other Neotropical ecoregions where palm-dwelling bugs of the genus *Rhodnius* [79] are major vectors of *T*. *cruzi*.

## Supporting information

S1 Fig Location of the study municipality (Araguatins) in Tocantins state, Brazil. The dark-green shade shows the approximate extent of Amazonian broadleaf forests, and the yellow shade to Cerrado savannas (see https://ecoregions2017.appspot.com/). Modified from Brito et al. [14] (https://doi.org/10.1371/journal.pntd.0006035.g001).
(TIF)

S1 Dataset Distance sampling of adult *Attalea* palms: Raw data. (CSV)

S2 Dataset *Rhodnius* spp. in adult *Attalea* palms: Raw data. (XLSX)

S3 Dataset *Attalea* palms studied using systematic sampling (this study) *vs*. “haphazard” palm selection (ref. [17]): Raw data. (XLSX)

S1 Text Detecting *Trypanosoma cruzi* in *Attalea*-dwelling *Rhodnius* spp.: Procedures, protocols, and results. (PDF)

S1 Table *Attalea* palm infestation by *Rhodnius* spp.: Exploratory generalized linear models (binomial distribution, logit link function). (PDF)

S2 Table Density of *Rhodnius* spp. colonies in *Attalea* palm crowns: Exploratory generalized linear models (zero-inflated; count submodels with Poisson distribution and log link function). (PDF)

S3 Table Systematically-sampled *Attalea* palms (present study) differ from those studied previously using “haphazard” palm selection (ref. [17]): Generalized linear models run on S3 Dataset. (PDF)
