# Supplementary figures and images for: Deforestation effects on Attalea palms and their resident Rhodnius, vectors of Chagas disease, in eastern Amazonia
Source: PLoS One. 2021 May 20;16(5):e0252071. doi: 10.1371/journal.pone.0252071 (PMC8136634; doi:10.1371/journal.pone.0252071)

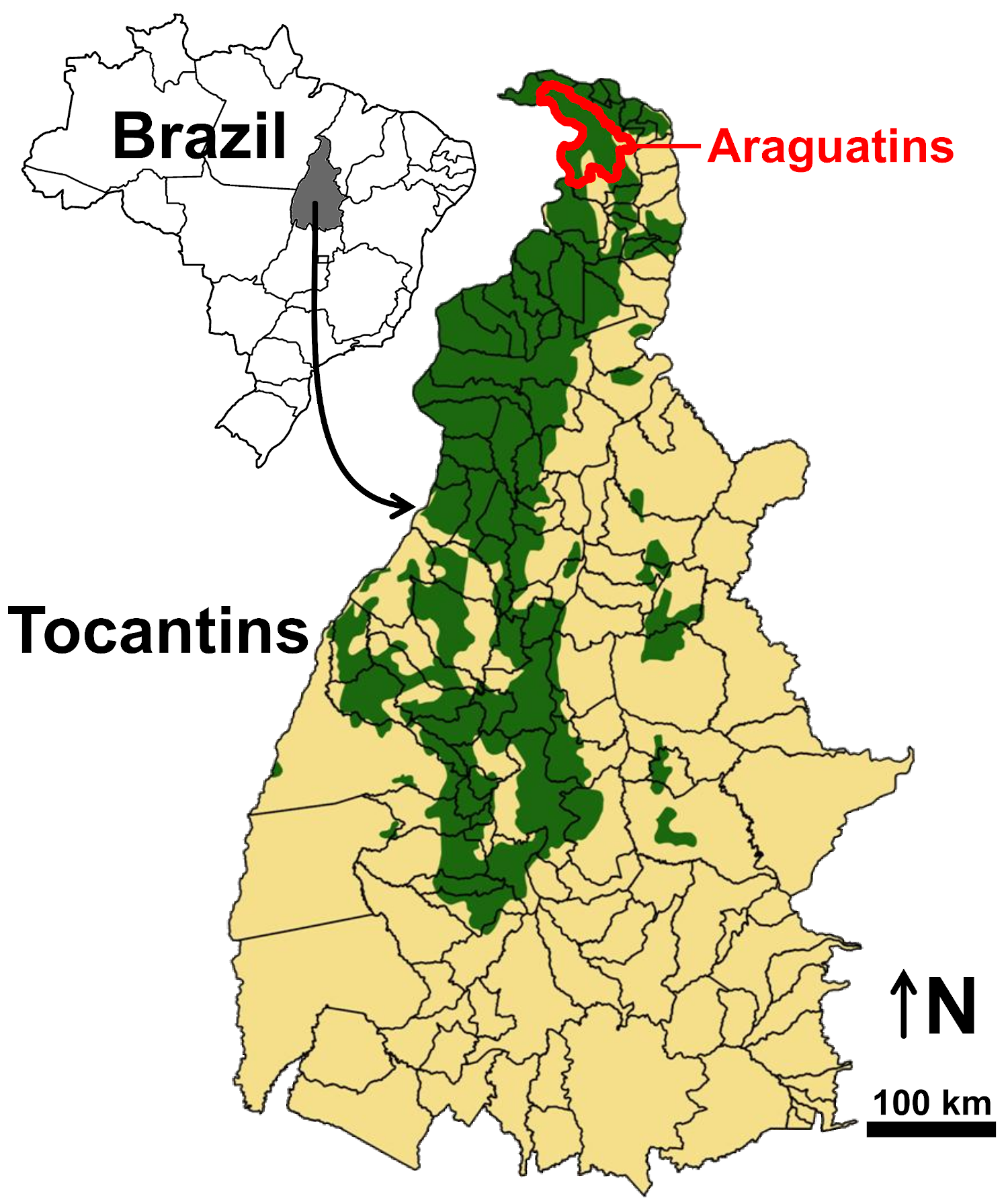

Supplement: S1 Fig — The dark-green shade shows the approximate extent of Amazonian broadleaf forests, and the yellow shade to Cerrado savannas (see https://ecoregions2017.appspot.com/). Modified from Brito et al. [14] (https://doi.org/10.1371/journal.pntd.0006035.g001). (TIF) [file pone.0252071.s001.tif]
