# Supplementary material for: Deforestation effects on Attalea palms and their resident Rhodnius, vectors of Chagas disease, in eastern Amazonia
Source: PLoS One. 2021 May 20;16(5):e0252071. doi: 10.1371/journal.pone.0252071 (PMC8136634; doi:10.1371/journal.pone.0252071)
Supplement: S1 Text — (PDF) [file pone.0252071.s005.pdf]

S1 Text

Detecting *Trypanosoma cruzi* in *Attalea*-dwelling *Rhodnius* spp.: procedures, protocols, and results

Bugs. We used the 33 bugs caught during the field activities described in the main body of the paper and 11 further bugs caught in palms of the same areas and transects in a second field trip. Note that these 11 bugs were not included in our main analyses on bug presence and abundance in palms.

DNA extraction and quantitative PCR. We used the Biopur Mini Spin Plus kit (Biometrix, Curitiba, Brazil), following the manufacturer's recommendations, to extract DNA from bug abdomens; DNA was quantified with a NanoVue Plus spectrophotometer (General Electric). We ran quantitative PCR (qPCR) assays to amplify the repetitive region of the *T. cruzi* nuclear satellite DNA as described in [S1]. Briefly, we used Ndao et al.'s [S2] primers (5'TGCACTCGGCTGATCGTTT; 5'ATTCCTCCAAGCAGCGGATA) in a mix reaction (final volume 20 µl) of 1× Power SYBR Green PCR Master Mix (Applied Biosystems), 0.2 µM of each primer, and 2 µl DNA template (10 ng). Reactions were run in duplicate in 96-well plates under the following conditions: 50°C for 2 min, 95°C for 10 min, and 40 cycles of 95°C for 15 sec, 60°C for 45 sec, and 72°C for 10 sec. Duplicate positive and negative controls plus blank controls were included in each qPCR run. Positive controls consisted of DNA extracted from a *T. cruzi* culture (Berenice strain), negative controls consisted of DNA extracted from uninfected, laboratory-reared *Rhodnius neglectus* and *R. nasutus*, and blank controls contained no DNA.

Estimating bug infection frequency. We scored each individual qPCR assay as yielding a 'detection' (coded '1'; *vs*. 'non-detection', coded '0') when we recorded amplification of the target with cycle threshold Ct ≤ 40.0 and a melting temperature

(Tm) between 81.1°C and 82.6°C. Acknowledging that our qPCR may not be 100% sensitive [S1], we used a single-species, single-season site-occupancy model [S3] to analyze our replicate *T. cruzi* detection/non-detection data – which are summarized in the Table below.

S1 Text Table. Detection/non-detection of *Trypanosoma cruzi* in 44 palm-dwelling *Rhodnius* spp.: results of duplicate quantitative PCR (qPCR) assays

Sample	qPCR_1	qPCR_2	Frequency
Field-caught bugs from *Attalea* palms	0	0	39
	1	0	1
	0	1	2
	1	1	2
Positive controls (*T. cruzi* DNA)	1	1	4
Negative controls (DNA from lab-reared, uninfected bugs)	0	0	4
Blank controls (no DNA)	0	0	2

qPCR_1 and qPCR_2, first and second quantitative-PCR replicates
qPCR results coded '1' = 'detection' and '0' = 'non-detection'

As can be seen in the Table, we detected *T. cruzi* DNA in five bugs (11.4%, or 0.114); all controls yielded results as expected – detections in positive controls and non-detections in negative and blank controls. Infected bugs were each caught in a different palm standing on disturbed landscapes – four palms in cattle pasture (all nymphs; one stage I, one stage II, and two stage III) and one in young secondary forest (one stage II nymph). None of the four bugs (all stage-II nymphs) caught in old-growth forest (two palms) yielded *T. cruzi* detections. Our sparse data did not allow us to confidently model infection heterogeneity across landscape classes. A simple site-occupancy model estimates mean bug-infection frequency at $\Psi = 0.118$ (95% confidence interval [CI] 0.050–0.256) for all landscapes, and mean qPCR sensitivity at $p = 0.80$ (CI 0.50–0.94) for each qPCR run.

Although clearly limited, these data (i) show that *T. cruzi* was circulating among its vectors (including young, wingless nymphs) in the crowns of *Attalea* palms of our

study sites, and (ii) suggest that such circulation might have been more intense in disturbed landscapes (with *T. cruzi* detected in 5/16 = 31.3% of infested palms) than in better-preserved old-growth forests (with no infection detected in the two palms that yielded bugs). This adds some (admittedly thin) evidence for the suggestion, based on the increase in palm infestation frequency and bug density shown in the main body of the paper, that deforestation increases the risk of contact between humans and infected vectors in eastern Amazonia.

References

S1. Minuzzi-Souza TTC, Nitz N, Cuba CAC, Hagström L, Hecht MM, Santana C, et al. Surveillance of vector-borne pathogens under imperfect detection: lessons from Chagas disease risk (mis)measurement. Sci Rep. 2018; 8:151.

S2. Ndao, M. Diagnosis of parasitic diseases: old and new approaches. Interdiscip Perspect Infect Dis. 2009; 2009:278246.

S3. MacKenzie DI, Nichols JD, Lachman GB, Droege S, Royle JA, Langtimm CA. Estimating site occupancy rates when detection probabilities are less than one. Ecology. 2002; 83:2248–2255.
