# Supplementary material for: Deforestation effects on Attalea palms and their resident Rhodnius, vectors of Chagas disease, in eastern Amazonia
Source: PLoS One. 2021 May 20;16(5):e0252071. doi: 10.1371/journal.pone.0252071 (PMC8136634; doi:10.1371/journal.pone.0252071)
Supplement: S1 Table — (PDF) [file pone.0252071.s006.pdf]

S1 Table. *Attalea* palm infestation by *Rhodnius* spp.: exploratory generalized linear models (binomial distribution, logit link function)

Model	AIC	Term	Estimate (SE)	CI lower	CI upper
Null model	135.6	Intercept	−2.68 (0.24)	−3.16	−2.20
Locality	132.4	Intercept	−3.37 (0.46)	−4.26	−2.48
		Locality L2	Ref.	-	-
		Locality L1	1.17 (0.54)	0.11	2.23
Landscape	106.4	Intercept	−4.45 (0.71)	−5.85	−3.06
		Old-growth forest	Ref.	-	-
		Cattle pasture	3.52 (0.78)	1.98	5.06
		Young secondary forest	1.51 (0.93)	−0.30	3.32
Palm stem height	128.5	Intercept	−2.92 (0.30)	−3.50	−2.34
		Stem height* (+1.4 m)	0.75 (0.26)	0.25	1.26
Palm organic score	135.1	Intercept	−2.73 (0.25)	−3.23	−2.23
		Organic score* (+0.8 units)	0.33 (0.20)	−0.05	0.72
Full model	90.3	Intercept	−6.17 (0.96)	−8.05	−4.28
		Locality L2	Ref.	-	-
		Locality L1	1.59 (0.66)	0.30	2.88
		Old-growth forest	Ref.	-	-
		Cattle pasture	4.06 (0.87)	2.36	5.77
		Young secondary forest	2.31 (1.00)	0.35	4.26
		Stem height* (+1.4 m)	0.98 (0.37)	0.26	1.69
		Organic score* (+0.8 units)	0.61 (0.32)	−0.01	1.23

AIC, Akaike's information criterion score; SE, standard error; CI lower and CI upper, lower and upper limits of the 95% confidence interval

*Continuous variables standardized to mean 0.0 and SD 1.0; effect estimates therefore correspond to an increase of 1 SD in the covariate value (indicated in parentheses)
