# Supplementary material for: Deforestation effects on Attalea palms and their resident Rhodnius, vectors of Chagas disease, in eastern Amazonia
Source: PLoS One. 2021 May 20;16(5):e0252071. doi: 10.1371/journal.pone.0252071 (PMC8136634; doi:10.1371/journal.pone.0252071)
Supplement: S2 Table — (PDF) [file pone.0252071.s007.pdf]

S2 Table. Density of *Rhodnius* spp. colonies in *Attalea* palm crowns: exploratory generalized linear models (zero-inflated; count submodels with Poisson distribution and log link function)

Model	AIC	Term	Estimate (SE)	CI lower	CI upper
Null model	182.4	Intercept	0.31 (0.24)	−0.16	0.78
Locality	178.4	Intercept	−0.85 (0.52)	−1.87	0.18
		Locality L2	Ref.	-	-
		Locality L1	1.34 (8.54)	0.28	2.39
Landscape	163.3	Intercept	−2.53 (0.59)	−3.70	−1.37
		Old-growth forest	Ref.	-	-
		Cattle pasture	3.01 (0.60)	1.82	4.19
		Young secondary forest	1.07 (0.75)	−0.40	2.53
Palm stem height	181.5	Intercept	−0.21 (0.37)	−0.94	0.51
		Stem height* (+1.4 m)	0.53 (0.29)	−0.03	1.09
Palm organic score	176.1	Intercept	−0.27 (0.31)	−0.87	0.33
		Organic score* (+0.8 units)	0.91 (0.28)	0.36	1.45
Full model	147.9	Intercept	−4.54 (0.77)	−6.06	−3.02
		Locality L2	Ref.	-	-
		Locality L1	1.10 (0.51)	0.11	2.10
		Old-growth forest	Ref.	-	-
		Cattle pasture	3.24 (0.62)	2.02	4.46
		Young secondary forest	1.56 (0.76)	0.07	3.04
		Stem height* (+1.4 m)	0.55 (0.24)	0.08	1.03
		Organic score* (+0.8 units)	0.70 (0.21)	0.28	1.12

AIC, Akaike's information criterion score; SE, standard error; CI lower and CI upper, lower and upper limits of the 95% confidence interval

*Continuous variables standardized to mean 0.0 and SD 1.0; effect estimates therefore correspond to an increase of 1 SD in the covariate value (indicated in parentheses)
