# Supplementary material for: Deforestation effects on Attalea palms and their resident Rhodnius, vectors of Chagas disease, in eastern Amazonia
Source: PLoS One. 2021 May 20;16(5):e0252071. doi: 10.1371/journal.pone.0252071 (PMC8136634; doi:10.1371/journal.pone.0252071)
Supplement: S3 Table — (PDF) [file pone.0252071.s008.pdf]

S3 Table. Systematically-sampled *Attalea* palms (present study) differ from those studied previously using "haphazard" palm selection (ref. [17]): generalized linear models run on S3 Dataset.

Dependent variable	Distribution	Link	Term	Estimate (SE)	CI lower	CI upper
Palm stem height	Gaussian	Identity	Intercept	6.23 (0.10)	6.03	6.43
			Present study	−1.48 (0.14)	−1.76	−1.20
Palm organic score	Gaussian	Identity	Intercept	1.74 (0.35)	1.67	1.80
			Present study	−1.00 (0.05)	−1.10	−0.91
Observed infestation	Binomial	Logit	Intercept	−1.50 (0.16)	−1.82	−1.18
			Present study	−1.18 (0.29)	−1.75	−0.60
Observed bug density	Poisson*	Log	Intercept	0.69 (0.11)	0.47	0.91
			Present study	−0.88 (0.28)	−1.43	−0.32

AIC, Akaike's information criterion score; SE, standard error; CI lower and CI upper, lower and upper limits of the 95% confidence interval

*Zero-inflated model with a Poisson distribution for the count submodel
